# Outcome of Allogeneic Stem Cell Transplantation Following Reduced-Intensity Conditioninig Regimen in Patients With Idiopathic Myelofibrosis: the G.I.T.M.O. Experience

**DOI:** 10.4084/MJHID.2010.010

**Published:** 2010-05-08

**Authors:** Francesca Patriarca, Andrea Bacigalupo, Alessandra Sperotto, Miriam Isola, Barbara Bruno, Maria Teresa van Lint, Anna Paola Iori, Paolo Di Bartolomeo, Maurizio Musso, Pietro Pioltelli, Giuseppe Visani, Pasquale Iacopino, Renato Fanin, Alberto Bosi.

**Affiliations:** 1 Division of Hematology and Cellular Therapies Unit ‘Carlo Melzi’, Dpt. of Morphological and Clinical Researches, University of Udine; 2 II Hematology Division, San Martino Hospital, Genova; 3 Statistics Institute, Dpt. of Morphological and Clinical Researches, University of Udine; 4 Hematology Division, ‘La Sapienza’ University, Roma; 5 Hematology Department, Pescara Hospital; 6 Oncohematology Unit, ‘La Maddalena’ Hospital, Palermo; 7 Internal Medicine and Hematology Division, ‘S. Gerardo de’ Tintori’ Hospital, Monza; 8 Division of Hematology, Pesaro Hospital; 9 Division of Hematology, Reggio Calabria Hospital; 10 Hematology Division, Azienda Ospedaliera Careggi, University of Firenze

## Abstract

**Background:**

Allogeneic stem cell transplantation (SCT) is a potentially curative treatment for myelofibrosis (MI), though limited by a high rate of transplant-related mortality (TRM). In the present study we evaluate the outcome of MI patients undergoing an allogenic SCT after reduced intensity conditioning (RIC) regimens, and the impact of prognostic factors.

**Design and methods:**

Fifty two patients were transplanted in 26 Italian centres between 1998 and 2006. We analyzed the influence of patient and disease clinical features before SCT and of transplant procedures on TRM and overall survival (OS) by means of univariate and multivariate analyses.

**Results:**

At SCT, median age was 52,5 years (32–68) and 89% of the patients had an intermediate or high Dupriez score. Conditioning regimens were based on fludarabine plus busulphan in 27% of patients, thiotepa plus cyclophosphamide in 46% and miscellaneous drug combinations in the other 27% of cases. Stem cells came from matched sibling donors for 75% of the patients and mismatched sibling or unrelated donors for the remaining 25%. The cumulative incidence of engraftment at day 90 after transplant was 83% (95% CI, 0.87–0.97). The estimated 1-year TRM was 30%. The estimated 3-year event-free-survival (EFS) and OS after hematopoietic SCT was 44% and 38% respectively. In multivariate analysis, an higher leukocyte count and circulating blasts in the peripheral blood before SCT significantly reduced EFS and OS respectively.

**Interpretation and conclusions:**

We conclude that the extension of the disease before transplantation based on the presence of circulating blasts and high leukocyte counts significantly affected the outcome after HSCT

## Introduction:

Myelofibrosis (MI) is a clonal hematopoietic stem cell disorder that is clinically characterized by progressive anemia, marked splenomegaly, extramedullary hematopoiesis, constitutional symptoms and a significant risk of evolution into acute leukaemia. MI can appear as a primitive or idiopathic disorder or, less frequently, as a secondary complication of essential thrombocythemia or polycythemia vera, with a clinical presentation and course similar to the idiopathic form. The disease affects mainly elderly people, with a median age at diagnosis of about 65 years. It is a heterogeneous disorder in term of presentation and evolution, with a median overall survival (OS) varying between 2 and 15 years, depending on the presence or absence of clinically defined prognostic factors. Adverse prognostic factors for survival have included advanced age, marked anemia, leukocytosis or leukopenia, abnormal karyotype, constitutional symptoms and presence of circulating blasts Moreover, the prognostic value of cytogenetic abnormalities, increased number of circulating CD34+ cells in peripheral blood and JAK2 mutational status has been also evaluated. The available prognostic score systems are mainly based on clinical variables. The most widely used is the Dupriez score^1^, which is based on hemoglobin level and leukocyte count. The Mayo Clinic Group tried to improve the Lille score by adding thrombocytopenia and monocytosis.^2^ The International Working Group for Myelofibrosis Research and Treatment (IWGMRT) recently proposed a new scoring system analyzing the largest patient population and recognized 5 main unfavourable variables which were: age > 65 years, presence of constitutional symptoms, and circulating blasts cells ≥ 1%, anemia and leucocytosis.^3^ All these prognostic systems could clearly separate intermediate or high risk patients (with a median survival ranging between 1 and 4 years) from patients with a favourable prognosis (median survival of 8–10 years).

Drug therapy is aimed at alleviating the symptoms, and has not been shown to improve survival. Allogeneic hematopoietic stem cell transplantation (HSCT) is the only treatment with the potential for curing MI and has been performed in patients with unfavourable clinical variables for several years. The application of HSCT in MI is strongly limited by an unacceptable rate of transplantation-related toxicity occurring in patients receiving standard myeloablative conditioninig. In fact, transplant-related mortality (TRM) ranged from 25 to 48% in different studies.^4^–^9^ The discovery that allogeneic stem cell can engraft patients prepared with nonmyeloablative doses of radiochemotherapy has led to the rapid development of a variety of reduced-intensity conditioning (RIC) regimens that have been successfully applied to patients with MI who would not be candidate for standard myeloablative HSCT due to advanced age or co-morbidities. RIC regimens might theoretically be applied to a large number of patients with an older age and comorbidities, while maintaining the potential for eradicating the disease based on the graft-versus MI effect.^10^–^14^ Studies in small series of patients who underwent RIC HSCT demonstrated the feasibility of the procedure, and reported encouraging TRM rates below 15% and OS rates around 80%. However, the patients included were a few and the follow-up was still short. RIC regimens were heterogeneous and included fludarabine plus low dose TBI or alkylating agent (busulfan or melphalan).

We have already published the analysis of the data of the Gruppo Italiano Trapianto Midollo Osseo (G.I.T.M.O.) Registry regarding a total of 100 patients with MI, who underwent allogeneic HSCT in 26 different Italian Transplant Centres between 1986 and 2005.^15^ Herein we present a sub-analysis limited to the patients who had received RIC regimens in more recent years, with the aim of analysing clinical features of patients and transplants and identifying prognostic factors affecting the outcome after HSCT.

## Methods:

Data were collected in an XLS database and imported into Stata/SE 9.0 for Windows for the statistical analysis. The end-points were engraftment, acute and chronic graft-versus-host disease (GVHD), relapse, TRM, overall survival (OS) and event-free survival (EFS).

TRM was defined as death due to all causes not related to MI. OS was defined as the time (months) from date of transplant to either death or last observation. EFS was defined as the time from date of transplant to relapse, death or last observation. OS and EFS were described using the Kaplan-Meier approach. Analysis of survival was done using Cox proportional hazard models, after the proportional hazards assumption had been verified. All variables considered in univariate analysis as possible prognostic factors were: primary diagnosis (primary or secondary MI), Dupriez score at transplant (low, intermediate, or high), transplant time (before and after 2001), interval between diagnosis and transplantation (months), transfusions before transplant, hemoglobin levels, leukocyte cell counts, circulating blasts, karyotype, previous splenectomy, splenomegaly at transplantation, age at transplantation, drugs of the conditioning regimen (fludarabine plus busulphan or thiotepa plus cyclophosphamide or other miscellaneous regimens), source of stem cells (marrow or peripheral blood), acute GVHD (grade 0-I or grade II–IV), and chronic GVHD (absent or limited or extensive). Multivariate stepwise analyses included all variables significant at p≤0.10 in an univariate analysis. Retention in the stepwise model required the variable to be significant at p≤0.05 in a multivariate analysis.

## Results:

A total of 52 patients were transplanted between January 1998 and December 2006. (**table 1 and 2**). Median time between diagnosis and HSCT was 13 months (1–161). At the time of HSCT, median age was 52,5 years (32–68), 89% of the patients had a Dupriez score ≥ 1, 45% had previously received red cells transfusions, 27% had blasts in the peripheral blood, 22 % had an abnormal karyotype, 51% had splenomegaly, 43% underwent splenectomy before HSCT. Conditioning regimens were based on fludarabine plus busulphan in 27% of patients, thiotepa plus cyclophosphamide in 46% and miscellaneous drug combinations in the other 27% of cases. Stem cells came from matched sibling donors for 75% of the patients and mismatched sibling or unrelated donors for the remaining 25%. Thirty-five per cent received bone marrow and 65% peripheral blood stem cells. Median time of follow-up after HSCT was 13 months for the whole population and 34 months for surviving patients. Eighty-three per cent achieved full engrafment. Eighteen patients developed acute GVHD grades II to IV for a cumulative incidence of 38% by day 100 after transplantation (95% CI 0.9–1.3). Chronic GVHD occurred in 20 patients for a cumulative incidence of 50% at 2 years (95% CI 0.4–0.8): it was limited in 14 patients and extensive in 6 patients. One-year TRM was 30%. The cumulative incidence of relapse was 19% at 1 year.

The estimated 3-year EFS and OS were respectively 44% and 38%. In univariate analysis factors associated with an higher TRM rate were: an older transplant date (1998–2000 vs 2001–2006: HR=0.25; CI_95%_[0.09;0.68]; p=0.007) and presence of grade II–IV acute GVHD (HR 2.42; CI_95%_[0.92–6.38]; p=0.073), The final survival model did not show any significant prognostic factor for TRM. Prognostic factors that were significantly (p≤0.10) associated with EFS in the univariate proportional hazards model were: transplant time (1998–2000 vs 2001–2006: HR=0.46; CI_95%_[0.19;1.15]; p=0.098), leukocyte count before transplantation (HR=1.00002; CI_95%_ [1.000002:1.000032]; p=0.028) and presence of chronic extensive GVHD (HR=0.27; CI_95%_ [0.06;1.26]; p=0.095). In multivariate analysis only an higher leukocyte count before HSCT was significantly associated with an unfavourable EFS (HR=1.000028; CI_95%_ [1.000011:1.000045]; p=0.001). In univariate analysis factors that affected OS were: presence of blasts in the peripheral blood before transplantation (HR=3.37; CI_95%_[0.36;2.39]; p=0.020), an higher leukocyte count before transplantation (HR=1.00002; CI_95%_ [1.000002:1.000035]; p=0.032), an older transplant date (1998–2000 vs 2001–2006: HR=0.32; CI_95%_ [0.21;0.82]; p=0.018). In multivariate analysis circulating blasts in the peripheral blood before transplantation significantly reduced OS (HR=3.356; CI_95%_[1.21;9.34]; p=0.020).

## Discussion:

We recently reported data on a population of 100 patients with MI who underwent allogeneic HSCT in 26 transplant centers that are part of the GITMO in a 20-year period between 1986 and 2006 and we retrospectively analyzed the influence of patient and disease clinical features before SCT and of transplant procedures on TRM and OS.^15^ We confirmed that MI remains a rare indication for SCT with the recruitment of about 15–17 cases per year since 2002 and observed a great heterogeneity in terms of conditioning regimens, GHVD prophylaxis and supportive measures. Although we observed a significative and progressive improvement of TRM after 1996, we could not recognized any significative difference in outcome either between patients treated with myeloablative versus RIC regimens or among those treated with different regimens. The lack of any association between intensity of conditioning or type of drugs included in the preparative regimen could be in part due to the great heterogeneity of transplant procedures. The present analysis is limited to the patients who had received RIC regimens, with the aim of identifying prognostic factors affecting the outcome after HSCT. This study confirms that outcome after RIC transplants has been progressively improved in the last decade. However, transplant toxicity is still a matter of concern, since we observed a 1- year TRM of 30% that is higher than that reported in previously published series after RIC transplants (4–6) and similar to TRM observed after myeloablative transplants.^10^–^14^ The extension of the disease before transplantation based on the presence of circulating blasts and high leukocyte counts significantly affected both EFS and OS after HSCT. These two parameters are both included in well-known prognostic scoring systems: the leukocyte count in the Dupriez score and the circulating blasts percentage in the IWGMRT one. These scoring systems, that were validated in non-transplanted patients, have demonstrated to impact the outcome after the transplant, too. In our series of patients we could not identify any transplant procedure (conditioning regimen or stem cell source or donor) influencing outcome, probably because of the small number of patients examined and the heterogeneity of chemotherapy and immunosuppressive drugs delivered. However, the most commonly used RIC regimen was the association of thiotepa and cyclophosphamide, that was administered to nearly half of the patients. Thiotepa is known to be a radiomimetic drug with a potent myeloablative activity associated with an immunosuppressive effect and minimal toxicity. The combination of thiotepa and cyclophosphamide was originally described for autologous transplants,^16^ then it was incorporated in the conventional preparative regimens for T-cell depleted allogeneic SCT from HLA matched and haploidentical sibling donors.^17^–^18^ The Genova group reported that thiotepa at the dose of 15 mg/kg and cyclophosphamide at the dose of 120–150 mg/kg produced complete engraftment and remission in 29 out of 31 patients, mostly with advanced disease.^19^ In a subsequent study, the same regimen with a 30% reduction of both drug doses was well tolerated by patients between 45 and 60 years with advanced leukemias, although half of the patients had persisting or relapsing disease.^20^ The incorporation of thiotepa in RIC regimens have represented an innovation in several Italian studies for different haematological diseases: thiotepa in association with cyclophosphamide and/or fludarabine followed by haematopoietic stem cells have demonstrated high feasibility and effectiveness in elderly patients with acute leukemias,^20^ in heavily pretreated relapsed or refractory lymphomas^21^ and in myelodisplastic syndromes.^22^

How do our study results compare with those already reported? **Table 3** summarized the clinical results of the studies published. Most of them were retrospective and include small series of patients. The largest series was reported by Kroeger on behalf of the European Group for Blood and Marrow Transplantation.^23^ The study was prospective and included 104 patients mainly with intermediate or high risk score who received a conditioning regimen based on fludarabine 180 mg/mq and busulfan 8 mg/kg i.v or 10 mg/Kg p.o and hematopoietic stem cells coming from sibling or unrelated donors. Engraftment was 99%; 1-year TRM was 16% and was significantly increased in patients older than 50 years, in cases with intermediate and high-risk MI and after transplants from mismatched donors. Five-year OS was 67% and 5 –year EFS was 51%. Relapse rate was higher in splenectomized patients and if disease duration prior transplant was > 24 months. Moreover, Kroeger reported that this conditioning regimen induced a JAK-2 negativity in 78% of patients carrying the V617-JAK2 mutation before transplant^24^ and produced a rapid regression of bone marrow fibrosis in 59% of patients al day +100 and in 100% of patients at day +360.^25^ At present, the busulfan-fludarabine regimen could be considered as the RIC regimen that has been tested on the largest patient population and demonstrated the best results in terms of feasibility and clinical, molecular and histological responses.

The largest single center experience was reported by Bacigalupo et al,^26^ concerning 46 patients who had received a RIC regimen based on thiotepa and cyclophosphamide. TRM was 24% and 5-year OS was 45% in the whole population, but it ranged from 8% to 77% according to the risk category. One of the most important contributions of these recent studies is the identification of the prognostic factors that influence the outcome after HSCT. Among the transplant-related variables, transplants from unrelated donors had a significative worse OS in comparison with sibling transplants in the Italian series^15^ and in the Genova study,^26^ while in the German study^23^ only mismatched unrelated donors had an unfavourable impact on OS. Among the disease-related variables, Dupriez score identified significative differences in the outcome after SCT in the German study,^23^ while Bacigalupo^26^ recognized large splenomegaly and higher than 20 red cell transfusions before transplant as powerful unfavourable predictors for OS. Role of splenectomy is still a controversial issue. Patients with large splenomegaly at transplant can have a slower granulocyte and platelet recovery, an higher risk of graft failure or persistence of splenomegaly for several months after transplant.^27^–^28^ However, splenectomy is associated of a 25% rate of bleeding and thrombotic complications and 6% perioperative deaths in patients with myelofibrosis.^29^ The increased risk of relapse after transplant in splenectomized patients observed in the German study was not confirmed in other series of patients. Since splenectomy is commonly performed in patients with larger splenomegaly, it can be hypothesized that splenectomy could be an indirect sign of a greater extension of the myeloproliferative disease before transplant, that could be associated to an higher risk of relapse after HSCT.

## Conclusions:

In conclusion, these results indicate that allogeneic SCT may be an attractive treatment approach for patients with high-risk MI. Although the outcome has been significantly improved in the last decade due to the reduction of TRM, several issues such as the timing of HSCT, the choice of the conditioning regimen, the source of stem cells, and the donor type have not been fully understood. Prospective studies that will explore new transplant modalities with the aims of reducing TRM and allow MI long-term control are warranted in this setting. The GITMO will promote a prospective study comparing two RIC regimes, fludarabine-busulfan versus fludarabine-thiotepa in patients with high-risk MI.

## Figures and Tables

**Table 1:** Clinical and haematological characteristics of the patients at allergeneic stem cell transplantation.

**Total no. Patients**	52
***Age***	
Median (range)	52,5 (32–68)
***Diagnosis***	
Idiopathic myelofibrosis	36/44 (82%)
Secondary myelofibrosis	8/44(18%)
***Time diagnosis-SCT (months)***	
Median (range)	13 (1–162)
≤ 12	21/52 (40%)
13–35	14/52 (27%)
≥ 36	17/52 (33%)
***Dupriez score at transplant***	
Low	5/44 (11%)
Intermediate	30/44 (68%)
High	9/44 (21%)
***Previous transfusions***	18/40 (45%)
***Karyotype***	
Normal	28/36 (78%)
Abnormal	8/36 (22%)
***Previous splenectomy***	20/46 (43%)
***Splenomegaly at transplant***	22/43 (51%)
***Circulating blasts at transplant***	11/41 (27%)
***Hemoglobin level at transplant (g/dl)***	
Median (range)	9.1 (5.6–14.4)
***White cell count at transplant (x 10*^*6*^*/l)***	
Median (range)	7.7 (0.6–118)

**Table 2:** Transplant-related characteristics

***Year of the transplant***	
1998–2000	8/52 (15%)
2001–2006	44/52 (85%)
***Donor***	
HLA matched sibling	39/52 (75%)
Unrelated or HLA “mismatched” sibling	13/52 (25%)
***Source***	
Bone marrow	18/52 (35%)
Peripheral blood	34/52 (65%)
***Conditioning regimen***	
Fludarabine plus busulphan	14/52 (27%)
Thiotepa plus cyclophosphamide	24/52 (46%)
Fludarabine- 2 Gy TBI	4/52 (8%)
Fludarabine-melphalan	6/52 (11%)
Other	4/52 (8%)

**Table 3.** Summary of the previously reported clinical results of allogeneic stem cell transplantation following reduced-intensity-conditioning transplants in myelofibrosis.

	**Rondelli^10^**	**Synder12**	**Merup14**	**Kroger23**	**Bacigalupo26**	**Present GITMO series**
**N° pts**	21	9	10	103	46	52
**Median age**	54 (27–68)	54 (46–68)	40 (5–63)	51 (24–67)	55 (32–68)	52 (32–68)
**RIC Conditioning**	Fluda + busulfan Thiotepa+cy Fluda+ melphalan Fluda + TBI	Fluda+ Melphalan Fluda + TBI	Fluda+ busulfan Fluda+Cy+ melphalan	Fluda + busulfan	Thiotepa + cy ±melphalan	Fluda + busulfan Thiotepa + cy
**Donor REL/UNREL**	19/2	2/7	20/7	33/70	32/14	39/13
**Median follow-up survivors**	31 months	32 months	50 months	33 months	39 months	34 months
**1 y-TRM %**	9%	44%	29%	16%	24%	30% (at 1 y)
**3y- OS %**	78% (at 2 y)	56%	70%	67% (at 5 y)	45% (at 5 y)	44% (at 3 y)
**3 y -relase**	9%	0%	Not evaluable	22 %	19%	19%
